# STAC3 incorporation into skeletal muscle triads occurs independent of the dihydropyridine receptor

**DOI:** 10.1002/jcp.26767

**Published:** 2018-08-02

**Authors:** Marta Campiglio, Mehmet M. Kaplan, Bernhard E. Flucher

**Affiliations:** ^1^ Department of Physiology Medical University, Innsbruck Innsbruck Austria

**Keywords:** DHPR, RyR1, skeletal muscle excitation‐contraction coupling, STAC3

## Abstract

Excitation‐contraction (EC) coupling in skeletal muscles operates through a physical interaction between the dihydropyridine receptor (DHPR), acting as a voltage sensor, and the ryanodine receptor (RyR1), acting as a calcium release channel. Recently, the adaptor protein SH3 and cysteine‐rich containing protein 3 (STAC3) has been identified as a myopathy disease gene and as an additional essential EC coupling component. STAC3 interacts with DHPR sequences including the critical EC coupling domain and has been proposed to function in linking the DHPR and RyR1. However, we and others demonstrated that incorporation of recombinant STAC3 into skeletal muscle triads critically depends only on the DHPR but not the RyR1. On the contrary, here, we provide evidence that endogenous STAC3 incorporates into triads in the absence of the DHPR in myotubes and muscle fibers of dysgenic mice. This finding demonstrates that STAC3 interacts with additional triad proteins and is consistent with its proposed role in directly or indirectly linking the DHPR with the RyR1.

## 1 INTRODUCTION

Contraction of skeletal muscle fibers is controlled by internal calcium released from the sarcoplasmic reticulum (SR) in response to electrical excitation of the cell membrane. In this process, termed excitation‐contraction (EC) coupling, depolarization of the plasma membrane is sensed by the dihydropyridine receptor (DHPR or Ca_V_1.1), a voltage‐gated calcium channel acting as a voltage sensor, and directly communicated to the SR calcium release channel, the type 1 ryanodine receptor (RyR1). To accomplish this tight functional coupling, DHPRs in the t‐tubule membrane and RyR1s in the SR are regularly arranged in close proximity to each other in the junctional membranes of triads or peripheral couplings (Block, 1988; Paolini, Fessenden, Pessah, & Franzini‐Armstrong, 2004). The loss of either the DHPR α_1S_ or β_1a_ subunits or of the RyR1 causes failure of EC coupling and perinatal death of null‐mutant mice (Gregg et al., 1996; Schredelseker et al., 2005; Takeshima et al., 1994; Tanabe, Beam, Powell, & Numa, 1988). Therefore, these three triad proteins were traditionally considered to represent the essential EC coupling proteins.

Recently, the adaptor protein STAC3 (SH3 and cysteine‐rich domain 3) has been identified as an additional essential EC coupling protein. STAC3 is exclusively expressed in skeletal muscles, where it is localized in the triads. Knockout of STAC3 causes loss of EC coupling and paralysis in mice and fish, and human mutations of STAC3 cause the debilitating Native American myopathy (NAM; Horstick et al., 2013; Nelson et al., 2013). In triads of STAC3 knockout (STAC3‐KO) myotubes, the expression of the DHPR as well as its gating and calcium currents is reduced, whereas EC coupling is completely abolished (Linsley, Hsu, Groom, et al., 2017; Polster, Nelson, Olson, & Beam, 2016). In the absence of STAC3, the DHPR current properties resembled those previously described in dyspedic (RyR1^−/−^) myotubes, suggesting that also the retrograde coupling of RyR1 and DHPR is abolished in STAC3 knockout muscle (Polster et al., 2016). Finally, X‐ray structure analysis identified a functionally important interaction between the first SH3 domain of STAC3 and the critical EC coupling domain in the cytoplasmic II–III loop of the DHPR. Both the NAM mutation in the SH3‐1 domain of STAC3 and mutating critical residues in the II–III loop of the DHPR perturbed STAC3–DHPR binding and EC coupling (Wong King Yuen, Campiglio, Tung, Flucher, & Van Petegem, 2017). This combined evidence suggested the intriguing possibility that STAC3 might be the missing functional link between the DHPR and RyR1. In fact, this notion is supported by coimmunoprecipitation and mass spectrometry data showing STAC3 binding to both the DHPR and RyR1 (Horstick et al., 2013). However, all subsequent studies only confirmed tight interactions between STAC3 and the DHPR, but not with the RyR1. Most importantly, we and others demonstrated that fluorescently tagged STAC3 constructs heterologously expressed in dysgenic (Ca_V_1.1^−/−^) myotubes failed to be incorporated into triads and that restoration of STAC3 triad targeting required expression of a DHPR α_1_ subunit (Ca_V_1.1 or Ca_V_1.2; Campiglio & Flucher, 2017; Polster, Perni, Bichraoui, & Beam, 2015). These results indicated that the DHPR was essential for the incorporation of STAC3 into the triads and that apart from the DHPR no interaction partners existed that were capable of recruiting STAC3 into the triads.

Here, we report new findings conflicting with these earlier observations. Using a specific antibody, we localized endogenous STAC3 in the triad junctions of cultured myotubes as well as in muscle fibers of dysgenic mice. This unexpected finding indicates that the triad contains binding sites for STAC3 in addition to those identified in the DHPR and that this putative interaction with endogenous STAC3 is strong enough to resist competition by heterologously expressed STAC3 constructs. It also suggests that STAC3 does not merely function as chaperone for DHPR triad targeting but is independently targeted into the triad where it supports the functional incorporation of DHPRs into the EC coupling complex and possibly the functional coupling with the RyR1.

## 2 MATERIALS AND METHODS

### 2.1 Dysgenic myotube culture and transfection

Myoblasts of the dysgenic (Ca_V_1.1^−/−^) cell line GLT were cultured in Dulbecco's modified Eagle's medium supplemented with 10% fetal calf serum, 10% horse serum, 2 mM of glutamine, penicillin (10 units/ml), and streptomycin (10 μg/ml; Gibco, Thermo‐Fischer scientific, Waltham, MA) and maintained at 37°C in a humified environment with 10% CO_2_. For immunostaining, myotubes of the homozygous dysgenic (mdg/mdg) GLT cell line were plated on carbon and gelatin‐coated coverslips and switched to serum‐free medium after 48 hr to induce myoblast fusion. At the onset of myoblast fusion (DIV 4), they were transiently transfected with STAC3 constructs with and without Ca_V_1.2 using FugeneHD (Promega, Fitchburg, WI), according to the manufacturer's instructions.

### 2.2 tsA201 cell culture and transfection

tsA201 cells stably expressing β_3_ and α_2_δ‐1 were cultured as previously described (Ortner et al., 2017). Cells were transiently transfected with 0.25 μg of plasmid cDNA using FugeneHD (Promega) on the day of plating. Cells were replated 24 h after transfection onto 13 mm poly‐l‐lysine‐coated coverslips and kept at 30°C with 5% CO_2_ for 24 h until fixation.

### 2.3 Immunofluorescence staining and image processing

Paraformaldehyde‐fixed cultures were stained as previously described (Campiglio & Flucher, 2017). Primary antibodies were used as follows: rabbit polyclonal anti‐STAC3 (1:2,000 in myotubes, 1:5,000 in tsA201 cells; Proteintech Group, Rosemont, IL); mouse monoclonal anti‐β_1_ (1:2,000, cl. N7/18; NeuroMab/National Institute of Health NeuroMab Facility, Davis, CA); mouse monoclonal anti‐RyR (1:1,000, cl. 34C; Alexis Biochemicals, San Diego, CA); rabbit polyclonal anti‐RyR1 (1:2,000; Barone et al., 1998); mouse monoclonal anti‐GFP (1:2,000, cl.270F3; Synaptic Systems, Goettingen, Germany); rabbit polyclonal anti‐GFP (1:10,000; Molecular Probes, Eugene, OR); and rat monoclonal anti‐HA (1:1,000; Roche Diagnostics, Basel, Switzerland). Secondary antibodies used were as follows: goat anti‐mouse Alexa 594 (1:4,000) and Alexa 488 (1:2,000); goat anti‐rabbit Alexa 594 (1:4,000) and Alexa 488 (1:4,000); and goat anti‐rat Alexa 594 (1:4,000, all from Invitrogen, Carlsbad, CA). Samples were analyzed with an AxioImager microscope (Carl Zeiss, Jena, Germany) using a ×63 1.4 NA objective. Fourteen‐bit images were recorded with a cooled CCD camera (SPOT; Diagnostic Instruments, Sterling Heights, MI) and Metaview image‐processing software (Universal Imaging, West Chester, PA). Figures were arranged in Adobe Photoshop, and linear contrast adjustments were performed. Semiquantitative analysis of β_1a_ and STAC3 coclustering was performed as previously described in 90 myotubes of at least three independent experiments (Campiglio & Flucher, 2017). Results are expressed as mean ± *SEM*.

### 2.4 Diaphragm muscle immunostaining

Maintenance and handling of dysgenic (Ca_V_1.1^−/−^) mice (Tanabe et al., 1988) conformed to the guidelines of the European Community (86/609/EEC) and were approved by the Austrian Ministry of Science (BMWFW‐66.011/0002‐WF/V/3b/2015). Homozygous wildtype or dysgenic mice were obtained from heterozygous mating, and diaphragm muscle was dissected from E18.5 embryos because homozygous dysgenic mice are not viable. Ribcages with the diaphragm were fixed with 4% paraformaldehyde in 0.1 M of phosphate buffer (pH 7.2) for 30 min at room temperature. Diaphragms were dissected in phosphate‐buffered saline (PBS) and incubated in 0.1 M of glycine in PBS for 1 hr at room temperature, permeabilized, and blocked in PBS containing 1% bovine serum albumin, 5% normal goat serum, and 0.2% Triton X‐100 overnight at 4°C. Primary antibodies mouse anti‐RyR1 (1:500) and rabbit STAC3 (1:1,000) were applied overnight at 4°C. The muscle samples were then washed three times at 1 hr intervals and incubated with secondary anti‐mouse Alexa 594 (1:4,000) and anti‐rabbit Alexa 488 (1:4,000) for 2 hr at room temperature. Diaphragms were then flat‐mounted in Vectashield mounting medium. Images were captured on a Leica microsystems SP5 laser scanning confocal microscope equipped with a ×63, 1.4 numerical aperture (NA) oil‐immersion lens (Leica Microsystems, Wetzlar, Germany). Fluorescence was excited using the 488 and 561 nm laser lines and recorded at bandwidths of 493–556 nm (green channel) and 566–752 nm (red channel), respectively. Eight‐bit images with 1024 × 256 pixels were acquired at 400 Hz scan speed. Color overlays and fluorescence intensity plots were generated using Metamorph (Universal Imaging). The analysis was performed on five diaphragms from three litters.

### 2.5 Cloning procedures

All plasmids contain the pcDNA3 backbone, and the expression is under control of a CMV promoter. Cloning procedures for pc‐Ca_V_1.1 (NM_001101720), pc‐Ca_V_1.2 (X15539), pc‐STAC3‐GFP (NM_177707), pc‐STAC1‐GFP (NM_016853), and pc‐STAC2‐GFP (NM_146028) were previously described (Campiglio & Flucher, 2017; Neuhuber, Gerster, Mitterdorfer, et al., 1998).

#### 2.5.1 pc‐GFP‐STAC3

*pc‐GFP‐STAC3*. The coding sequence of STAC3 was isolated by polymerase chain reaction (PCR) using pc‐STAC3‐GFP as template, with the forward primer introducing a *Sal*I site and the reverse primer introducing an *Eco*RI site. The PCR fragment was then *Sal*I/*Eco*RI digested and introduced into the corresponding sites of GFP‐Ca_V_1.1 (Grabner, Dirksen, & Beam, 1998), yielding pc‐GFP‐STAC3.

#### 2.5.2 pc‐STAC3‐HA

The coding sequence of STAC3 was amplified by PCR using pc‐STAC3‐GFP as template. The forward primer was designed to introduce a *Kpn*I site, whereas the reverse primer introduced an HA tag and *Xho*I site. The STAC3‐HA fragment generated by PCR was then *Kpn*I/*Xho*I digested and introduced into the corresponding sites of pc‐STAC3‐GFP, yielding pc‐STAC3‐HA.

## 3 RESULTS

**Figure 1:**
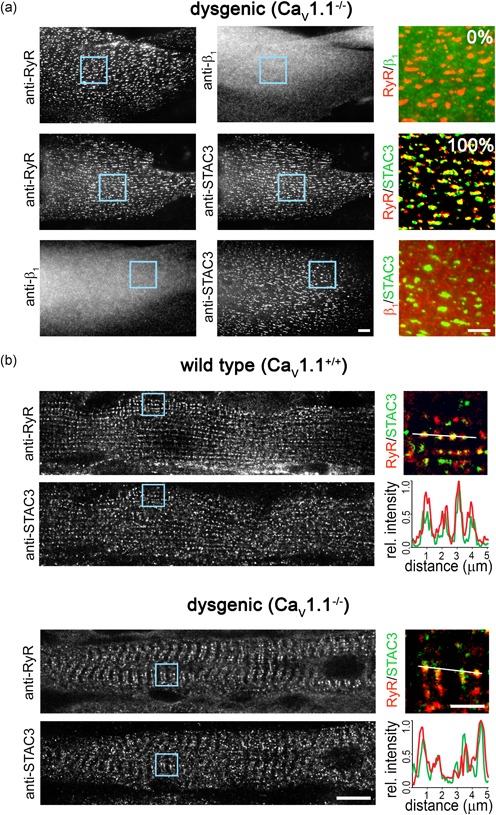
STAC3 localizes in the triads of dysgenic (Ca_V_1.1^−/−^) myotubes in vitro and in vivo. (a) Representative immunofluorescence images of dysgenic myotubes stained to label the ryanodine receptor (RyR), the DHPR β_1a_ subunit, and STAC3. Scale bar = 10 μm. The numbers indicate the percentage of myotubes in which β_1a_ or STAC3 colocalized with the RyR (*N* = 3, *n* = 90). Color overlays are 4× magnifications of the regions indicated by the blue squares. Scale bar = 5 μm. (b) Diaphragm muscle fibers of wildtype and dygenic mice, double‐labeled with anti‐RyR and anti‐STAC3. Scale bar = 10 μm. 4× magnification color overlays of STAC3 (green) and RyR (red). Scale bar = 2.5 μm. Fluorescence intensity scans along two sarcomeres of both wildtype (upper panel) and dysgenic (lower panel) muscles show peaks for STAC3 (green) and RyR1 (red) at exactly the same location consistent with their colocalization in the triads. DHPR, dihydropyridine receptor [Color figure can be viewed at wileyonlinelibrary.com]

Myotubes of dysgenic (Ca_V_1.1^−/−^) mice form triads and peripheral junctions containing RyR1, but lacking the DHPR. When double‐labeled with anti‐RyR1 and anti‐β_1a_, RyR1 is localized in a clustered distribution pattern, indicating its localization in the triads, whereas β_1a_ is evenly distributed throughout the cytoplasm because it requires the lacking DHPR α_1_ subunit for its own incorporation into the EC coupling apparatus (Figure 1a; Neuhuber, Gerster, Doring, et al., 1998).

Previously, we and others utilized C‐terminal GFP‐tags to analyze triad targeting and association with DHPR α_1_ subunits (Ca_V_1.1 or Ca_V_1.2) of STAC proteins in dysgenic myotubes. The use of tagged STAC constructs enabled us to analyze the stability of STAC‐DHPR complexes in living muscle cells and to apply the same antibody detection system for studying the properties of chimeric and mutated STAC proteins (Campiglio & Flucher, 2017). Similarly to the β_1a_ subunit, STAC3‐GFP remained diffusely distributed in the cytoplasm of dysgenic myotubes in the absence of a DHPR α_1_ subunit but adopted a clustered distribution and colocalized with RyR1 in the presence of Ca_V_1.1 or Ca_V_1.2 (Campiglio & Flucher, 2017). These findings were in agreement with similar observations by Polster et al. (2015) also using C‐terminally tagged STAC3 and together led to the conclusion that the incorporation of STAC3 into skeletal muscle triads requires the DHPR.

Here, we used immunostaining to analyze the distribution of endogenous STAC3 in dysgenic myotubes. The rabbit STAC3 antibody has been previously used for Western blot analysis (Cong et al., 2016). We established antibody specificity in a cellular context in tsA201 cells transiently transfected with either STAC1‐GFP, STAC2‐GFP, or STAC3‐GFP, where the anti‐STAC3 antibody specifically labeled STAC3‐GFP‐transfected cells (Supporting Information Figure S1).

In dysgenic myotubes lacking the DHPR, we expected to obtain the same diffused distribution with anti‐STAC3 labeling as previously observed with heterologous STAC‐GFP (Campiglio & Flucher, 2017). However, STAC3 immunolabeling of untransfected dysgenic myotubes resulted in a clustered distribution (Figure 1a). In all analyzed myotubes, the STAC3 clusters were colocalized with the RyR1, whereas the DHPR β_1a_ subunit was diffusely distributed, clearly indicating that endogenous STAC3 is localized in triads and peripheral junctions in the absence of the DHPR in dysgenic myotubes.

To examine whether this was also the case in skeletal muscle fibers in vivo, we repeated the RyR1–STAC3 double‐immunofluorescence staining in diaphragm muscle fibers of dysgenic mice at embryonic day E18½. Consistent with the observation in the cultured myotubes, STAC3 in dysgenic muscle fibers was colocalized with RyR1 in the triads, similarly to the wildtype controls (Figure 1b). Although wildtype muscles at E18½ are better differentiated than the quiescent dysgenic muscles, in both genotypes, STAC3 was organized in transverse double rows of clusters and colocalized with the RyR1. Together, the immunolocalization in dysgenic skeletal muscle triads in vitro and in vivo demonstrates that STAC3 incorporation into the EC coupling apparatus does not depend on the presence of the DHPR.

**Figure 2:**
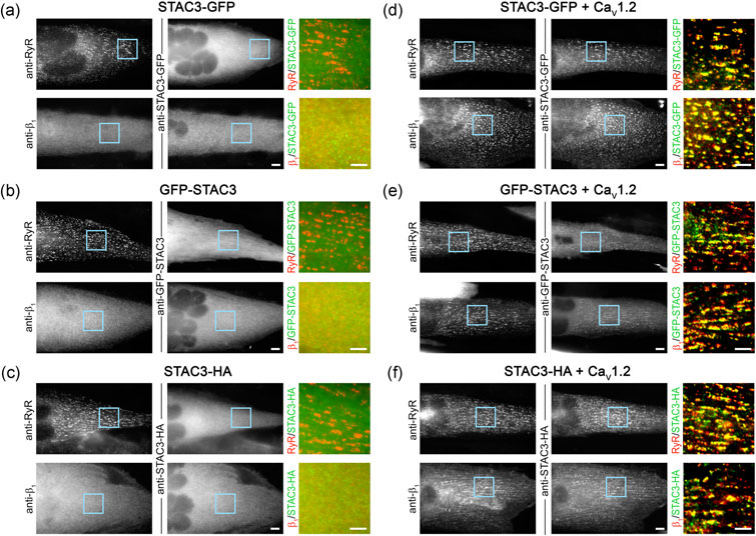
Failure of triad targeting of heterologously expressed STAC3 in dysgenic (Ca_V_1.1^−/−^) myotubes is independent of the orientation and size of the tag. (a–c) In the absence of a Ca_V_1 α_1_ subunit, triads form, as indicated by RyR1 clusters, but heterologously expressed STAC3‐GFP (a), GFP‐STAC3 (b), or STAC3‐HA (c) is diffusely localized in the cytoplasm, similarly to the distribution of the β_1a_ subunit. (d–f) Upon reconstituition with the DHPR Ca_V_1.2 subunit, STAC3‐GFP (d), GFP‐STAC3 (e), and STAC3‐HA (f) as well as the β_1a_ subunit associate with the DHPR complex and colocalize with RyR in the triads. Scale bar = 10 μm. Color overlays are 4× magnifications of the regions indicated by the blue squares. Scale bar = 5 μm. DHPR, dihydropyridine receptor [Color figure can be viewed at wileyonlinelibrary.com]

Why then GFP‐tagged recombinant STAC3 constructs were not observed in triads of dysgenic myotubes in previous studies? To examine the possible involvement of the tag in occluding recombinant STAC3‐GFP from the triads, we generated a STAC3 construct with a different orientation of the tag (GFP‐STAC3) and another construct with a smaller epitope tag (STAC3‐HA) and expressed them in dysgenic myotubes. Whereas all three STAC3 constructs were recruited to the triads when coexpressed with the Ca_V_1.2 α_1_ subunit, none of these recombinant STAC3 constructs was localized in the triads in the absence of a DHPR α_1_ subunit (Figure 2). Instead, all three constructs showed a diffused, cytoplasmic distribution similar to that of β_1a_. These controls indicate that neither the position of the GFP tag nor the tag size caused the occlusion of recombinant STAC3 from skeletal muscle triads.

Alternatively, association of endogenous STAC3 with hitherto unidentified binding sites in the triad may be strong enough to resist competition by heterologously expressed STAC3 constructs in the absence of the DHPR, whereas expression of the DHPR α_1_ subunit provides additional binding sites, which then can be occupied by recombinant STAC3 proteins. In light of this competition model, it is noteworthy to recall previous results showing that heterologous STAC3‐GFP associates better with Ca_V_1.2 (100% of the myotubes) than with Ca_V_1.1 (~60% of the myotubes; Campiglio & Flucher, 2017), as if the cardiac–neuronal DHPR α_1_ subunit had a higher affinity for STAC3 than that of its native skeletal muscle partner Ca_V_1.1. However, also when dysgenic myotubes were reconstituted with Ca_V_1.1 or Ca_V_1.2, the triads were equally well occupied with endogenous STAC3 (Supporting Information Figure S2). Thus, the lower efficacy of Ca_V_1.1 in recruiting STAC3‐GFP observed previously may reflect a higher occupancy of its binding sites with endogenous STAC3 due to the optimal assembly of all three components (Ca_V_1.1, STAC3, and RyR1) in the skeletal muscle EC coupling apparatus. Conversely, Ca_V_1.2, which is not directly associated with the RyR1 (Kasielke, Obermair, Kugler, Grabner, & Flucher, 2003; Takekura, 2004), may not be within reach of endogenous STAC3 in the EC coupling apparatus and therefore be better accessible for heterologous STAC3‐GFP. Thus, the finding that endogenous STAC3 colocalizes with the RyR1 in triads with and without the DHPR is consistent with its putative role in mediating the interaction between the DHPR and RyR1 in skeletal muscle EC coupling, and it provides reasonable explanations for previously unexplained observations.

## 4 DISCUSSION

The result presented here demonstrates that, unlike the DHPR β_1a_ subunit, endogenous STAC3 does not require a DHPR α_1_ subunit to localize in skeletal muscle triads (Figure 1). This contrasts previous observations where heterologously expressed STAC3 was not clustered in triads of DHPR‐null myotubes (Campiglio & Flucher, 2017; Polster et al., 2015). That lack of incorporation into the triads of heterologous STAC3 does not depend on tag size or orientation (Figure 2), suggesting that endogenous STAC3 in the triads prevents incorporation of heterologous STAC3 into the triadic complex in the absence of the DHPR. Consistent with this notion, Polster et al. (2018) recently reported that in the absence of endogenous STAC3, heterologously expressed neuronal STAC isoforms are incorporated in the triads of the myotubes. Yet, the same STAC isoforms failed to be incorporated in the triads of myotubes containing Ca_V_1.1 and endogenous STAC3 (Campiglio & Flucher, 2017). To conclusively demonstrate that in the absence of the DHPR endogenous STAC3 prevents triad incorporation of heterologously expressed STAC3, a STAC3–DHPR double null‐mutant muscle expression system will need to be established.

Because STAC3 had first been identified as an essential skeletal muscle EC coupling protein, it was hypothesized to function either as the physical link between the DHPR and the RyR1 or like an auxiliary subunit of either one of the two calcium channels (Horstick et al., 2013; Nelson et al., 2013). Because previous results suggested that triad targeting of heterologous STAC3 required the DHPR but not the RyR1, STAC3 was believed to act like an auxiliary subunit of the DHPR and it was even termed DHPR ε subunit (Bannister, 2016). Indeed, studies performed on STAC3‐null myotubes found that the absence of STAC3 compromised the trafficking, stability, and function of the DHPR in the membrane, similarly to what had been observed in the absence of β_1a_ (Linsley, Hsu, Groom, et al., 2017; Polster et al., 2015, 2016). However, a more recent study revealed a substantial difference between STAC3 and the β_1a_ subunit. Whereas in the absence of the β_1a_ triad, targeting of the DHPR failed, this was not the case in the absence of STAC3 (Linsley, Hsu, Wang, et al., 2017). Our present finding that STAC3 is located in the triads independent of the DHPR further emphasizes the distinct roles of β_1a_ and STAC3 in trafficking and stabilizing the DHPR, respectively. STAC3 and the DHPR are targeted and incorporated in triads independent of each other, although interactions with the DHPR are sufficient to recruit STAC3 to the membrane. Conversely, STAC3 may contribute to the stabilization of the DHPR in the triad. Our present findings clearly indicate that STAC3 binds another triad protein apart from the DHPR and thus are consistent with the proposed role of STAC3 as the physical link between the DHPR and the RyR1.

**Figure 3:**
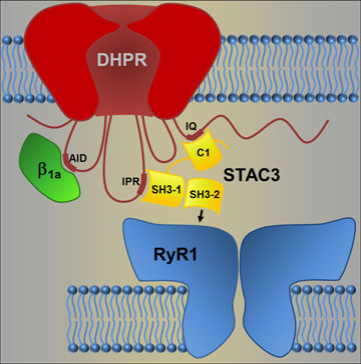
Model of the STAC3 interactions in the skeletal muscle excitation‐contraction (EC) coupling apparatus. STAC3 has been demonstrated to establish two discrete interactions with the dihydropyridine receptor (DHPR): its C1 domain interacts with the IQ domain in the C‐terminus, and the SH3‐1 domain interacts with the IPR motif in the II–III loop of the DHPR. Here, we provide evidence of a third, hitherto unidentified, interaction, which anchors STAC3 in the triad junctions independent of the DHPR. We propose that it is established by the SH3‐2 domain. Because of the crucial role of STAC3 in EC coupling and because no additional proteins are necessary to restore voltage‐induced calcium release in heterologous cells, STAC3 may thus directly couple the DHPR with the RyR1. RyR1, ryanodine receptor [Color figure can be viewed at wileyonlinelibrary.com]

STAC3 contains a C1 domain and tandem SH3 domains (SH3‐1 and SH3‐2; Figure 3). Recently, we determined that the C1 domain and the tandem SH3 domains engage in separate interactions with the DHPR: one critical for the targeting of heterologous STAC3 to the triads and for modulating the inactivation of Ca_V_1.2 calcium currents (Campiglio et al., 2018) and the other involved in EC coupling (Wong King Yuen et al., 2017). More specifically, the crystal structure of the tandem SH3 domains in a complex with the II–III loop of the DHPR revealed that this interaction mostly involves the SH3‐1 domain with a smaller contribution of the SH3‐2 domain, leaving the canonical binding surface of the SH3‐2 domain available for additional interactions (Wong King Yuen et al., 2017).

Because STAC3 colocalizes with the RyR1 in the absence of the DHPR and is critical for voltage‐induced calcium release by the RyR1, it is possible that the two proteins interact with each other. This interaction could be direct, which is consistent with their copurification in a proteomics study (Horstick et al., 2013), or indirect, as suggested by fluorescence recovery after photobleaching (FRAP) experiments in tsA201 cells (Polster et al., 2015). Recently, the group of Kurt Beam reconstituted skeletal muscle‐like DHPR‐RyR1 coupling in tsA201 cells with only five proteins—the DHPR α_1_ and β_1a_ subunits, STAC3, RyR1, and junctophilin 2 (Perni, Lavorato, & Beam, 2017)—thus excluding the possibility that further proteins might be necessary to mediate the interaction between DHPR and RyR1. Our current finding that STAC3 can associate with the RyR1 in triads lacking the DHPR α_1_ and β_1a_ subunits now eliminates the remaining conceptual obstacle for the otherwise likely model that STAC3 mediates skeletal muscle EC coupling by directly linking the voltage sensor (DHPR) with the SR calcium release channel (RyR1).

## CONFLICTS OF INTEREST

The authors declare no financial or nonfinancial competing interests.

## Supporting information

Supporting information

## References

[jcp26767-bib-0001] Bannister, R. A. (2016). Bridging the myoplasmic gap II: More recent advances in skeletal muscle excitation‐contraction coupling. Journal of Experimental Biology, 219(Pt 2), 175–182. 26792328 10.1242/jeb.124123

[jcp26767-bib-0002] Barone, V. , Bertocchini, F. , Bottinelli, R. , Protasi, F. , Allen, P. D. , Franzini Armstrong, C. , … Sorrentino, V. (1998). Contractile impairment and structural alterations of skeletal muscles from knockout mice lacking type 1 and type 3 ryanodine receptors. FEBS Letters, 422(2), 160–164. 9489997 10.1016/s0014-5793(98)00003-9

[jcp26767-bib-0003] Block, B. A. (1988). Structural evidence for direct interaction between the molecular components of the transverse tubule/sarcoplasmic reticulum junction in skeletal muscle. Journal of Cell Biology, 107(6 Pt 2), 2587–2600. 2849609 10.1083/jcb.107.6.2587 PMC2115675

[jcp26767-bib-0004] Campiglio, M. , Costé de bagneaux, P. , Ortner, N. J. , Tuluc, P. , Van Petegem, F. , & Flucher, B. E. (2018). STAC proteins associate to the IQ domain of CaV1.2 and inhibit calcium‐dependent inactivation. Proceedings of the National Academy of Sciences of the United States of America, 115, 1376–1381. 29363593 10.1073/pnas.1715997115 PMC5819422

[jcp26767-bib-0005] Campiglio, M. , & Flucher, B. E. (2017). STAC3 stably interacts through its C1 domain with CaV1.1 in skeletal muscle triads. Scientific Reports, 7, 41003. 28112192 10.1038/srep41003 PMC5253670

[jcp26767-bib-0006] Cong, X. , Doering, J. , Mazala, D. A. G. , Chin, E. R. , Grange, R. W. , & Jiang, H. (2016). The SH3 and cysteine‐rich domain 3 (Stac3) gene is important to growth, fiber composition, and calcium release from the sarcoplasmic reticulum in postnatal skeletal muscle. Skeletal Muscle, 6, 17. 27073615 10.1186/s13395-016-0088-4 PMC4828897

[jcp26767-bib-0007] Grabner, M. , Dirksen, R. T. , & Beam, K. G. (1998). Tagging with green fluorescent protein reveals a distinct subcellular distribution of L‐type and non‐L‐type Ca2+ channels expressed in dysgenic myotubes. Proceedings of the National Academy of Sciences of the United States of America, 95(4), 1903–1908. 9465115 10.1073/pnas.95.4.1903 PMC19211

[jcp26767-bib-0008] Gregg, R. G. , Messing, A. , Strube, C. , Beurg, M. , Moss, R. , Behan, M. , … Powers, P. A. (1996). Absence of the beta subunit (cchb1) of the skeletal muscle dihydropyridine receptor alters expression of the alpha 1 subunit and eliminates excitation‐contraction coupling. Proceedings of the National Academy of Sciences of the United States of America, 93(24), 13961–13966. 8943043 10.1073/pnas.93.24.13961 PMC19477

[jcp26767-bib-0009] Horstick, E. J. , Linsley, J. W. , Dowling, J. J. , Hauser, M. A. , McDonald, K. K. , Ashley‐Koch, A. , … Kuwada, J. Y. (2013). Stac3 is a component of the excitation‐contraction coupling machinery and mutated in Native American myopathy. Nature Communications, 4, 1952. 10.1038/ncomms2952 PMC4056023 23736855

[jcp26767-bib-0010] Kasielke, N. , Obermair, G. J. , Kugler, G. , Grabner, M. , & Flucher, B. E. (2003). Cardiac‐type EC‐coupling in dysgenic myotubes restored with Ca2+ channel subunit isoforms alpha1C and alpha1D does not correlate with current density. Biophysical Journal, 84(6), 3816–3828. 12770887 10.1016/S0006-3495(03)75109-1 PMC1302963

[jcp26767-bib-0011] Linsley, J. W. , Hsu, I. U. , Groom, L. , Yarotskyy, V. , Lavorato, M. , Horstick, E. J. , … Kuwada, J. Y. (2017). Congenital myopathy results from misregulation of a muscle Ca2+ channel by mutant Stac3. Proceedings of the National Academy of Sciences of the United States of America, 114(2), E228–E236. 28003463 10.1073/pnas.1619238114 PMC5240691

[jcp26767-bib-0012] Linsley, J. W. , Hsu, I. U. , Wang, W. , & Kuwada, J. Y. (2017). Transport of the alpha subunit of the voltage gated L‐type calcium channel through the sarcoplasmic reticulum occurs prior to localization to triads and requires the beta subunit but not Stac3 in skeletal muscles. Traffic, 18(9), 622–632. 28697281 10.1111/tra.12502 PMC5569907

[jcp26767-bib-0013] Nelson, B. R. , Wu, F. , Liu, Y. , Anderson, D. M. , McAnally, J. , Lin, W. , … Olson, E. N. (2013). Skeletal muscle‐specific T‐tubule protein STAC3 mediates voltage‐induced Ca2+ release and contractility. Proceedings of the National Academy of Sciences of the United States of America, 110(29), 11881–11886. 23818578 10.1073/pnas.1310571110 PMC3718085

[jcp26767-bib-0014] Neuhuber, B. , Gerster, U. , Doring, F. , Glossmann, H. , Tanabe, T. , & Flucher, B. E. (1998). Association of calcium channel alpha1S and beta1a subunits is required for the targeting of beta1a but not of alpha1S into skeletal muscle triads. Proceedings of the National Academy of Sciences of the United States of America, 95(9), 5015–5020. 9560220 10.1073/pnas.95.9.5015 PMC20205

[jcp26767-bib-0015] Neuhuber, B. , Gerster, U. , Mitterdorfer, J. , Glossmann, H. , & Flucher, B. E. (1998). Differential effects of Ca2+ channel beta1a and beta2a subunits on complex formation with alpha1S and on current expression in tsA201 cells. Journal of Biological Chemistry, 273(15), 9110–9118. 9535900 10.1074/jbc.273.15.9110

[jcp26767-bib-0016] Ortner, N. J. , Bock, G. , Dougalis, A. , Kharitonova, M. , Duda, J. , Hess, S. , … Striessnig, J. (2017). Lower affinity of isradipine for L‐type Ca2+ channels during Substantia Nigra dopamine neuron‐like activity: Implications for neuroprotection in Parkinson's disease. Journal of Neuroscience, 37(28), 6761–6777. 28592699 10.1523/JNEUROSCI.2946-16.2017 PMC6596555

[jcp26767-bib-0017] Paolini, C. , Fessenden, J. D. , Pessah, I. N. , & Franzini‐Armstrong, C. (2004). Evidence for conformational coupling between two calcium channels. Proceedings of the National Academy of Sciences of the United States of America, 101(34), 12748–12752. 15310845 10.1073/pnas.0404836101 PMC515124

[jcp26767-bib-0018] Perni, S. , Lavorato, M. , & Beam, K. G. (2017). De novo reconstitution reveals the proteins required for skeletal muscle voltage‐induced Ca(2+) release. Proceedings of the National Academy of Sciences of the United States of America, 114(52), 13822–13827. 29229815 10.1073/pnas.1716461115 PMC5748219

[jcp26767-bib-0019] Polster, A. , Nelson, B. R. , Olson, E. N. , & Beam, K. G. (2016). Stac3 has a direct role in skeletal muscle‐type excitation‐contraction coupling that is disrupted by a myopathy‐causing mutation. Proceedings of the National Academy of Sciences of the United States of America, 113(39), 10986–10991. 27621462 10.1073/pnas.1612441113 PMC5047181

[jcp26767-bib-0020] Polster, A. , Nelson, B. R. , Papadopoulos, S. , Olson, E. N. , & Beam, K. G. (2018). Stac proteins associate with the critical domain for excitation‐contraction coupling in the II‐III loop of CaV1.1. Journal of General Physiology, jgp.201711917. 10.1085/jgp.201711917 PMC5881444 29467163

[jcp26767-bib-0021] Polster, A. , Perni, S. , Bichraoui, H. , & Beam, K. G. (2015). Stac adaptor proteins regulate trafficking and function of muscle and neuronal L‐type Ca2+ channels. Proceedings of the National Academy of Sciences of the United States of America, 112(2), 602–606. 25548159 10.1073/pnas.1423113112 PMC4299259

[jcp26767-bib-0022] Schredelseker, J. , Di Biase, V. , Obermair, G. J. , Felder, E. T. , Flucher, B. E. , Franzini‐Armstrong, C. , & Grabner, M. (2005). The beta 1a subunit is essential for the assembly of dihydropyridine‐receptor arrays in skeletal muscle. Proceedings of the National Academy of Sciences of the United States of America, 102(47), 17219–17224. 16286639 10.1073/pnas.0508710102 PMC1288016

[jcp26767-bib-0023] Takekura, H. (2004). Differential contribution of skeletal and cardiac II‐III loop sequences to the assembly of dihydropyridine‐receptor arrays in skeletal muscle. Molecular Biology of the Cell, 15(12), 5408–5419. 15385628 10.1091/mbc.E04-05-0414 PMC532020

[jcp26767-bib-0024] Takeshima, H. , Lino, M. , Takekura, H. , Nishi, M. , Kuno, J. , Minowa, O. , … Noda, T. (1994). Excitation‐contraction uncoupling and muscular degeneration in mice lacking functional skeletal muscle ryanodine‐receptor gene. Nature, 369(6481), 556–559. 7515481 10.1038/369556a0

[jcp26767-bib-0025] Tanabe, T. , Beam, K. G. , Powell, J. A. , & Numa, S. (1988). Restoration of excitation‐contraction coupling and slow calcium current in dysgenic muscle by dihydropyridine receptor complementary DNA. Nature, 336(6195), 134–139. 2903448 10.1038/336134a0

[jcp26767-bib-0026] Wong King Yuen, S. M. , Campiglio, M. , Tung, C. C. , Flucher, B. E. , & Van Petegem, F. (2017). Structural insights into binding of STAC proteins to voltage‐gated calcium channels. Proceedings of the National Academy of Sciences of the United States of America, 114(45), E9520–E9528. 29078335 10.1073/pnas.1708852114 PMC5692558

