# Supplementary material for: STAC3 incorporation into skeletal muscle triads occurs independent of the dihydropyridine receptor
Source: J Cell Physiol. 2018 Aug 2;233(12):9045–51. doi: 10.1002/jcp.26767 (PMC6334165; doi:10.1002/jcp.26767)
Supplement: Supplementary file 1 — Supporting information [file JCP-233-9045-s001.docx]

**Supplementary figures**


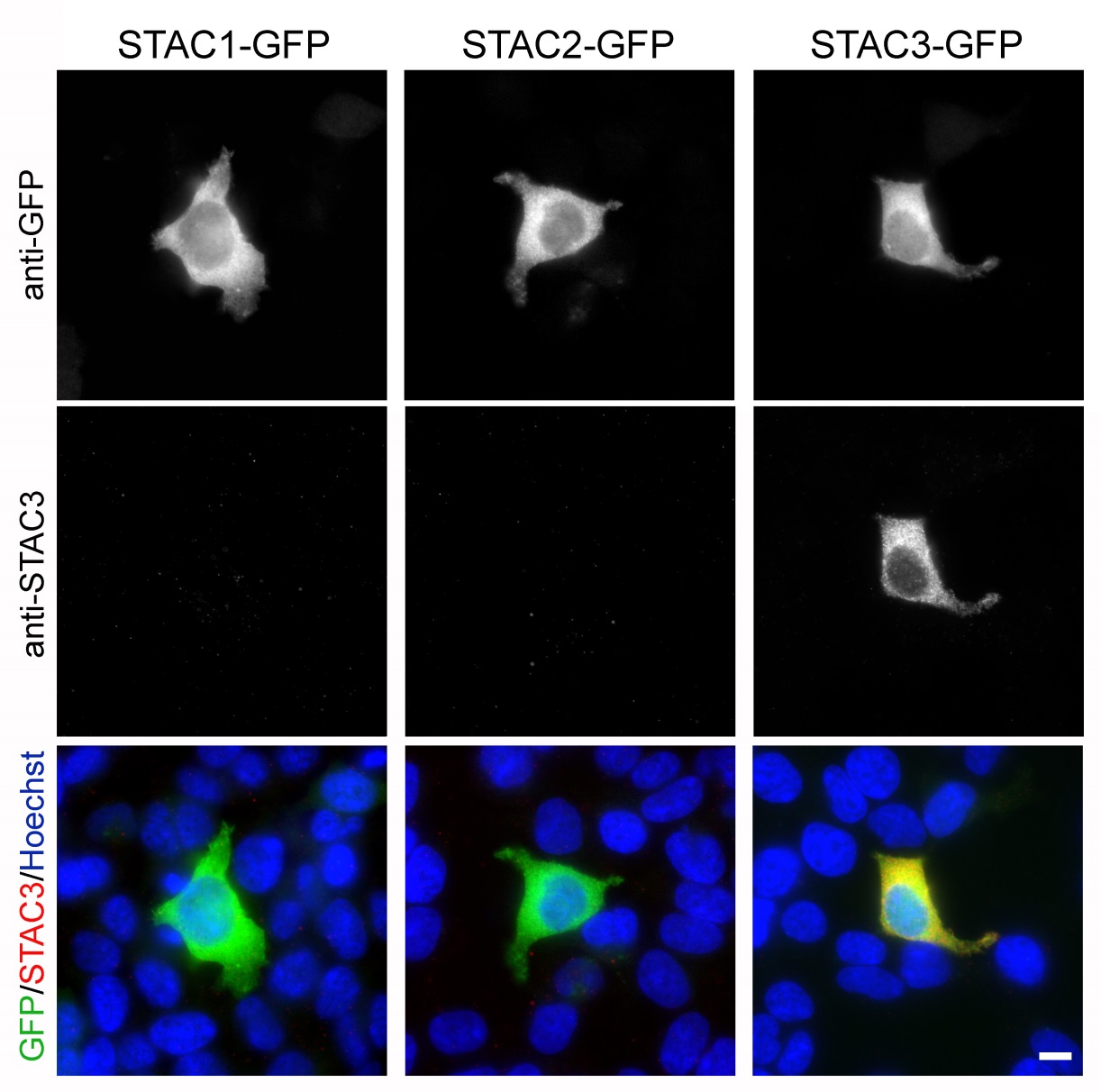


**Figure S1. Specificity of the STAC3 antibody.** The polyclonal antibody against STAC3 was tested in tsA201 cells transfected with STAC1-GFP (left), STAC2-GFP (middle), or STAC3-GFP (right). The intrinsic fluorescence of the GFP tag allowed the detection of the transfected GFP-tagged STAC subunits. The anti-STAC3 antibody specifically stained STAC3-GFP transfected cells (right), but not STAC1-GFP (left) or STAC2-GFP (middle) expressing cells. Non-transfected neighboring cells (the nuclei of which are stained by the Hoechst dye) also showed no reaction with the STAC3 antibody. Scale bar, 10 μm.


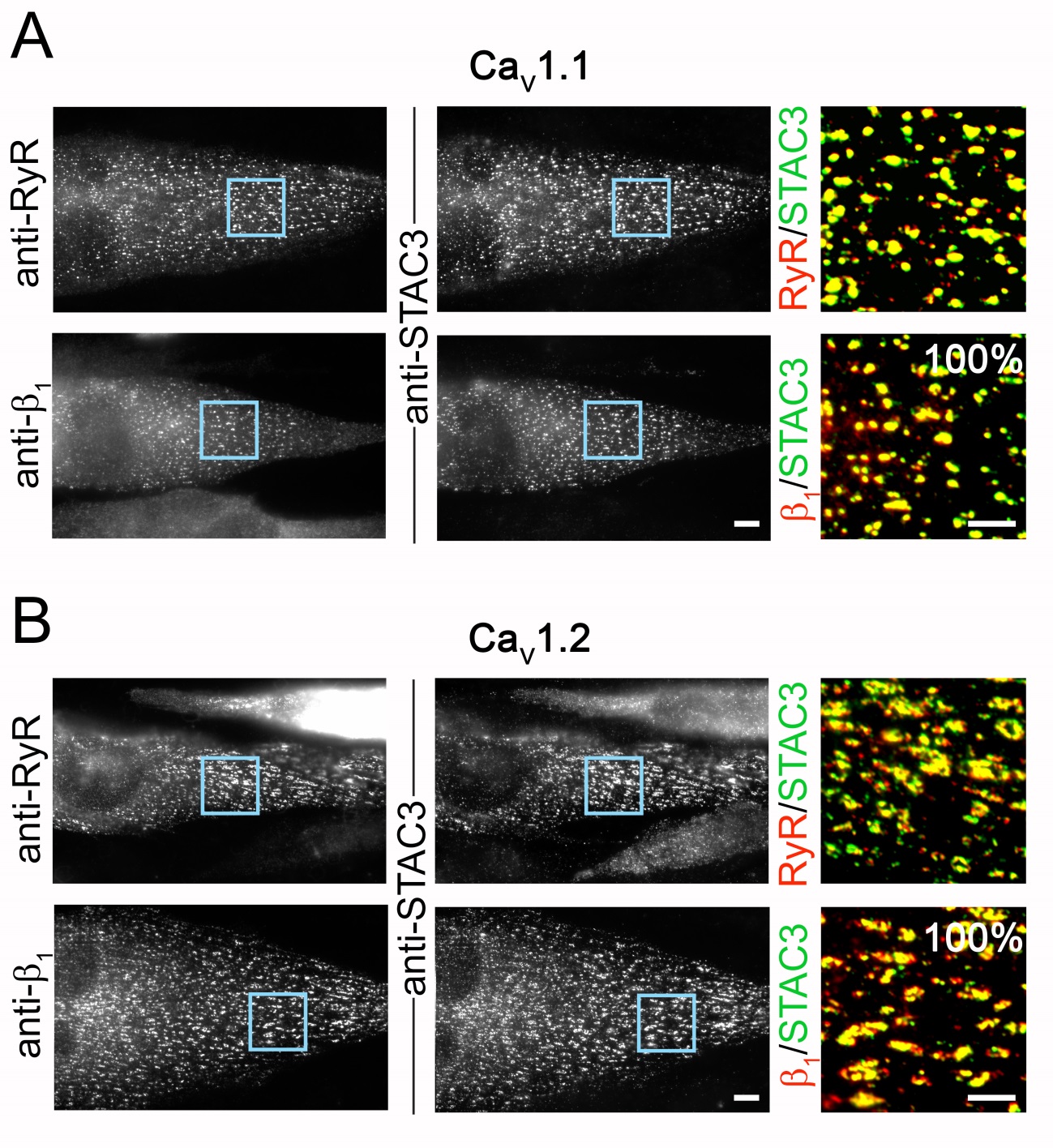


**Figure S2. Endogenous STAC3 localizes in the triads of dysgenic (Ca_V_1.1^-/-^) myotubes reconstituted with Ca_V_1.1 or Ca_V_1.2.** Representative immunofluorescence images of dysgenic myotubes reconstituted with either Ca_V_1.1 (**A**) or Ca_V_1.2 (**B**). Scale bar, 10 μm. Color overlays are 4X magnifications of the regions indicated by the blue squares. Scale bar, 5 μm. The numbers indicate the percentage of myotubes in which STAC3 colocalized with β_1_ (N=3, n=90). Note that the triads of all analyzed myotubes containing either Ca_V_1.1 or Ca_V_1.2 also contained endogenous STAC3.
